# Mind the gap: challenges and future directions for content-based image retrieval in clinical radiology

**DOI:** 10.3389/fradi.2026.1810708

**Published:** 2026-05-15

**Authors:** Erika Spada, Katharina Erb-Eigner, Aurélie Pahud de Mortanges, Mauricio Reyes, Alexander Pöllinger

**Affiliations:** 1 Medical Image Analysis Lab, ARTORG Center for Biomedical Engineering Research, University of Bern, Bern, Switzerland; 2 Department of Radiology, Charité–Universitätsmedizin Berlin, Corporate Member of Freie Universität Berlin and Humboldt-Universität Zu Berlin, Berlin, Germany; 3 Department of Diagnostic, Interventional and Pediatric Radiology, Inselspital, Bern University Hospital, Bern, Switzerland; 4 Department of Radiation Oncology, Inselspital, Bern University Hospital, University of Bern, Bern, Switzerland

**Keywords:** AI in radiology, clinical AI, content-based image retrieval, radiology image retrieval, trajectory retrieval

## Abstract

Artificial intelligence (AI) has become increasingly integrated into radiology across multiple domains, including image acquisition, interpretation, workflow optimization, and clinical decision support. Among these applications, advanced image retrieval systems have the potential to assist clinicians in identifying visually and clinically similar cases, as well as relevant prior studies. This article provides a comprehensive overview of content-based image retrieval (CBIR) and introduces the emerging paradigm of trajectory retrieval, which focuses on the longitudinal evolution of diseases over time. We discuss the clinical applications of these systems, including diagnostic decision support, workflow optimization, educational uses, and research cohort building, highlighting their potential to enhance diagnostic reasoning and reduce uncertainty in complex or rare cases. Despite substantial research progress, adoption in real-world clinical settings remains limited due to challenges such as data heterogeneity, privacy constraints, the need for multimodal integration, and difficulties in capturing temporal dynamics. Trajectory-based approaches, combined with multimodal data integration and human-in-the-loop feedback mechanisms, offer a promising path toward overcoming these barriers by aligning retrieval systems more closely with the longitudinal and holistic nature of clinical decision-making. By addressing these challenges, AI-powered radiology image retrieval has the potential to transform workflows, support more precise and confident diagnoses, and ultimately improve patient care outcomes.

## 1 Introduction to AI-based radiology image retrieval

AI-based radiology image retrieval refers to the use of artificial intelligence to identify prior imaging studies by querying large archives with an example image rather than textual keywords. A radiologist selects an image, or a specific region of interest, and the system searches for examinations that exhibit comparable imaging patterns. The goal is to provide access to clinically meaningful precedent cases that may support interpretation in routine practice. For retrieval systems to be genuinely useful, retrieved cases must be tied to a reliable diagnostic ground truth, ideally confirmed through histopathology, microbiology, genetic testing, or other accepted clinical standards, and supported by documented follow-up. Without this foundation, even the most visually similar case can become misleading rather than helpful.

### 1.1 Why radiologists need content-based image retrieval systems

Radiologists frequently face diagnostic uncertainty. Imaging findings may be subtle or atypical, and both common and rare diseases can present in unexpected ways. Even for experienced readers, interpreting imaging characteristics such as morphology or signal behavior is demanding, particularly as modern examinations generate hundreds to thousands of images under substantial time constraints.

In uncertain situations, radiologists often seek precedent cases by consulting textbooks, online resources, colleagues, or attempting manual Picture Archiving and Communication System (PACS) searches. However, modern repositories contain millions of examinations collected across institutions, modalities, and protocols, making manual searches unrealistic. Current PACS systems rely on keyword queries based on radiology reports, which are often incomplete or inconsistent and may overlook essential imaging details. As a result, radiologists often make decisions without access to visually similar prior cases, even when such cases exist in their own archives. This mismatch persists because radiology is inherently visual, yet search tools remain text based.

Content based image retrieval (CBIR) systems address this by analyzing the visual content of an image rather than relying on keywords. They allow radiologists to query an archive using an example image or a region of interest and then retrieve studies with similar imaging patterns. Modern AI-based systems can recognize shapes, textures, signal intensities, and spatial patterns in volumetric images and retrieve comparable cases, thereby providing an institutional visual “memory” grounded in image similarity (1).

By retrieving cases with verified diagnoses and outcomes, contemporary CBIR frameworks can support differential diagnosis, facilitate recognition of rare or atypical presentations, and increase diagnostic confidence (2–6). Benchmarking studies further indicate that new frameworks, particularly those based on deep learning and transformer architectures, can achieve clinically meaningful retrieval relevance across organs and modalities in realistic radiology workflows (2, 4, 5).

Importantly, these systems are not meant to replace radiologists, but they function as **decision support tools** (5–7).

### 1.2 Technical approaches

The technical foundation of radiology image retrieval has evolved from early semi-manual tools to sophisticated AI-driven methods.

CBIR systems perform two core tasks. First, they create *a feature representation* of an image by translating visual elements such as edges, shapes, textures, densities, or signal intensities into a mathematical. Then, this representation is used to *index and search* large image repositories to efficiently retrieve examinations that exhibit similar visual characteristics (8).

Early systems required substantial human input, with radiologists manually delineating regions of interest processed using basic computer vision algorithms (9). These approaches depended on manual annotations and handcrafted features (10, 11).

Over time, many techniques were explored for feature extraction, including histograms, wavelets and Fourier transforms, local binary patterns, and other texture descriptors (12). Other approaches have utilized pixel intensity, texture, and shape-based features (13), which were usually organized into three levels: primitive (low-level) features like brightness and texture, logical (medium level) features describing objects and spatial relationships, and abstract (semantic) features (14).

Despite their conceptual appeal, these traditional methods had major limitations: they required extensive manual engineering, were difficult to standardize, and often failed to generalize (10, 12, 15, 16).

A central limitation was the so-called **semantic gap**, referring to the disconnect between low-level computational features and the high-level concepts used by clinicians to describe disease (14). Traditional approaches with manually crafted features often failed to bridge this gap effectively (17, 18). In contrast, modern deep learning methods can automatically learn discriminative representations more closely aligned with clinical semantics (17). Convolutional neural networks and related architectures identify disease-relevant patterns without explicit feature engineering, markedly improving retrieval performance and reducing sensitivity to task-specific design choices (11, 12).

Some advanced retrieval systems combine images with accompanying text, such as radiology reports or clinical notes. This approach, often called *multimodal learning*, helps the model to learn how imaging patterns relate to diagnostic terminology. To achieve this, statistical and AI based methods learn joint representations in which related images and text are placed close together, allowing the system to retrieve cases that are both visually similar and clinically meaningful (19). Integrating these systems with clinical infrastructure, including PACS, has also been explored (20, 21).

Despite these advances, retrieval remains technically challenging: medical images differ fundamentally from natural images, and large-scale annotated radiology datasets remain scarce (17, 22). Furthermore, many retrieval concepts are often limited to particular modalities, organs, or diagnostic studies, making it difficult to transfer directly to other medical applications (14).

## 2 Clinical applications

Radiology image retrieval systems have a broad range of potential clinical applications that extend beyond purely technical image matching. The main clinical use cases are outlined below.

**1. Diagnostic Decision Support and Enhanced Clinical Confidence:** AI-powered retrieval systems can identify visually and semantically similar cases, helping radiologists, especially with rare or complex conditions, by providing precedents with known outcomes (2, 23). Access to precedent cases helps contextualize imaging findings, refine differential diagnoses, and increase diagnostic confidence (1, 3, 7).
**2. Medical Education:** CBIR systems support training by allowing residents and students to search using example images rather than keywords. When based on curated and validated cases, they function as dynamic teaching files that reflect real-world pattern recognition (24).
**3. Workflow Prioritization:** Retrieval systems can contribute to workflow management by identifying imaging patterns associated with urgent conditions, supporting case prioritization in high-volume clinical settings (4, 25).
**4. Reducing Radiologist Workload:** By providing rapid access to relevant reference cases, retrieval systems reduce the need for manual searches and reliance on memory, helping radiologists work more efficiently under increasing workload pressures (26).
**5. Clinical Research Cohort Building:** Content-based methods help researchers to quickly collect specific types of images, simplifying the creation of cohorts with defined imaging characteristics for clinical studies (27).
**6. Explainable AI Diagnosis:** Linking AI predictions to similar historical cases improves transparency and interpretability, allowing radiologists to assess automated outputs in a familiar clinical context (4, 28).
**7. Reducing Inter-observer Variability:** Reference cases with confirmed diagnoses help standardize interpretations among radiologists, addressing the high inter-observer variability that occurs when analyzing radiological images (29).
**8. Integration with Electronic Health Records:** Advanced implementations aim at integrating medical image retrieval with Electronic Medical Records (EMR) and Radiology Information Systems (RIS), creating comprehensive clinical information systems (30).
**9. Linking Images with Radiology Reports:** Analysis of large collections of radiology reports helps link imaging appearances to diagnostic terminology, improving retrieval relevance and clinical usefulness (31).

For illustration, Table 1 presents some representative CBIR examples per application.

**Table 1:** Illustrative clinical applications of content-based image retrieval (CBIR) in radiology.

Ref.	Clinical Scenario	Clinical Question	Retrieval Role	Modality	Target Anatomy/Pathology	AI Approach	Dataset	Key Findings
(7)	Diagnostic support	Diagnostic classification of indeterminate orbital masses	Retrieval of visually similar cases with known diagnoses	MRI	Orbital masses	Deep metric learning–based CBIR	142 MRIs (48 patients and 36 radiologists)	Improved diagnostic classification in observer study
(32)	Medical education	Diagnostic accuracy and confidence in interstitial lung disease	Retrieval of visually similar cases	CT	Interstitial lung disease	CBIR with pattern matching	28 cases, 4 readers	Learning effect observed especially for radiology residents
(25)	Workflow prioritization	Identification of clinically relevant tumor cases	Volumetric similarity–based case prioritization	CT, MRI	Solid tumors (colon, liver, lung, pancreas)	Context-aware 3D CBIR	601 volumetric scans (∼115,899 slices)	Effective case prioritization for oncologic workflow
(26)	Reducing workload	Interpretation of diffuse lung patterns	Retrieval of reference patterns during reporting	CT	Diffuse parenchymal lung disease	PACS-integrated CBIR	108 CT exams; 6,542 annotated patterns	31.3% reduction in reading time (*p* < 0.001); trend to higher accuracy
(22)	Explainable AI	Enhancing transparency and trust in deep CBIR through explainability analysis of retrieval features	Retrieval of similar CT liver cases with interpretable feature representations	CT	Liver	Self-supervised learning with domain-knowledge integration	Multi-institutional CT liver datasets	Improves performance and generalization while revealing feature extraction mechanisms
(33)	Reducing inter-reader variability	Assisting readers in UIP CT pattern classification	Retrieval of top 3 similar chest CT images with UIP classification	CT	Usual interstitial pneumonia (UIP) pattern	Deep learning CBIR	587 patients, 100 query cases, 9 readers	Improved diagnostic accuracy and inter-reader agreement for UIP pattern classification across experience levels
(34)	EHR-integrated decision support	COVID-19 diagnosis and prognosis	Retrieval of similar CXR with linked EHR data	Chest x-Rays	Lungs	Deep metric learning with spatial attention	18,055 CXR from 9 hospitals (USA, South Korea)	Combined image + EHR features improved 72 h intervention prediction
(31)	Image–report alignment	Association of imaging patterns with report language	Text-guided similarity retrieval	CT	Interstitial lung disease (17 subtypes)	Text-guided metric learning CBIR	1,093 CT scans (265 patients)	Improved retrieval relevance via report integration

For each clinical scenario, the table summarizes the role of retrieval, imaging modality, target condition, AI approach, dataset characteristics, and key findings from the cited study.

### 2.1 Clinical adoption gap

While AI in radiology has achieved broad clinical adoption, with 48% of European radiologists reporting active AI use (35), these tools focus on detection, classification, and reporting (36). No commercial CBIR system currently exists for clinical radiology. The few prototypes remain in academic settings (21). CBIR has advanced in digital pathology, where tools such as SMILY (37) and Yottixel (38) have demonstrated the feasibility of similarity-based search. Yet even in pathology, no tool has achieved widespread diagnostic deployment. Key barriers include the absence of curated databases with verified ground truth, the lack of standardized retrieval metrics, context-dependent similarity definitions, and unclear regulatory pathways for decision-support tools (36, 39).

## 3 Current challenges and future directions

**Figure 1:**
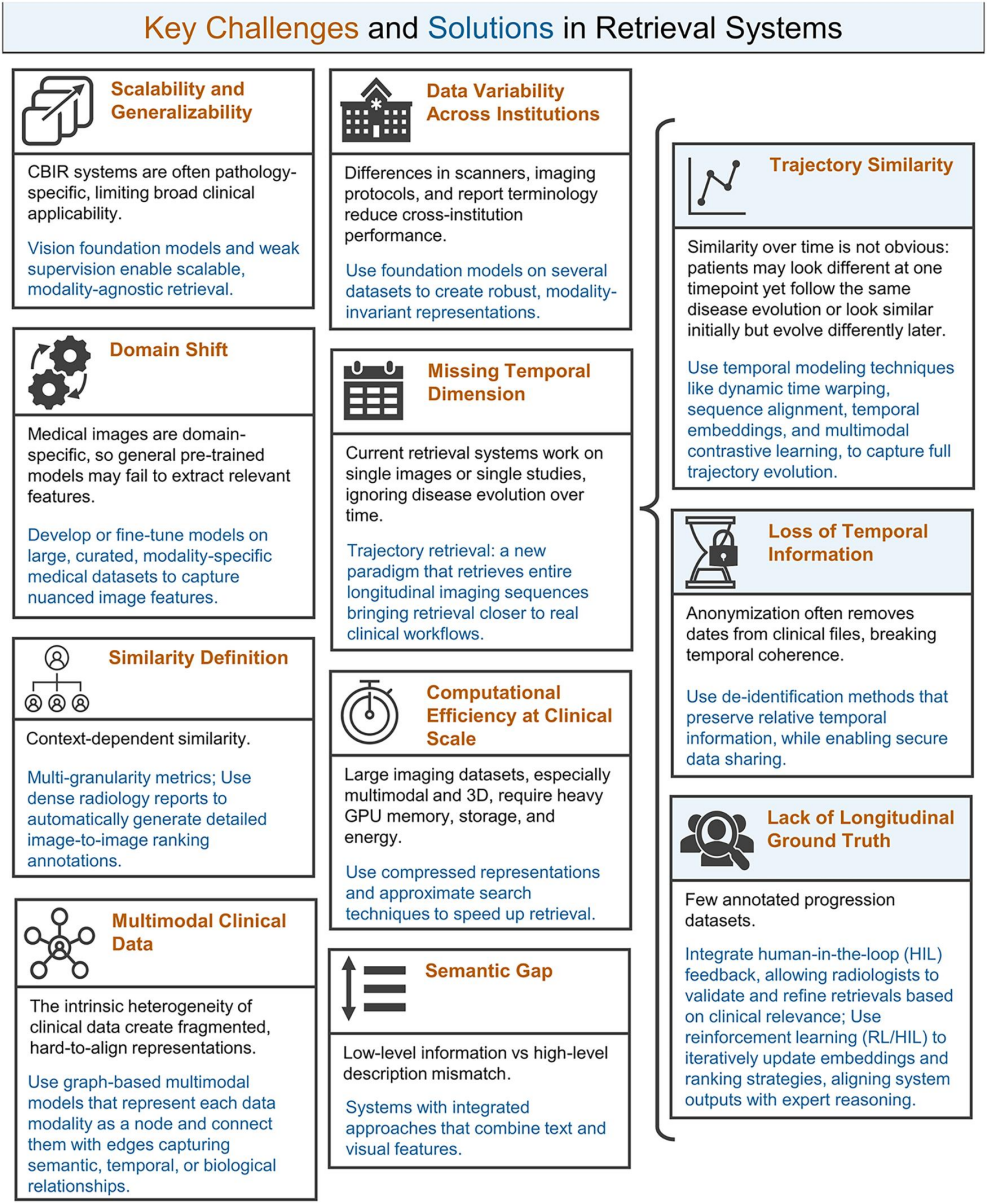
Key challenges in radiology image retrieval. Challenges are highlighted in ochre, while corresponding solutions are shown in blue. The right column represents the challenges arising from the introduction of the trajectory retrieval paradigm.

Despite substantial progress, several important challenges remain, as summarized in Figure 1, and are explained below.

### 3.1 Semantic gap

As mentioned before, a central issue is the semantic gap: computers extract low-level visual features such as shapes, textures, or intensities, while radiologists interpret high-level clinical concepts like “spiculated mass”. Bridging this gap requires combining visual features with textual information from radiology reports, linking image patterns to diagnostic terminology (40).

### 3.2 Medical domain shift

Medical images differ fundamentally from natural images used in standard computer vision. Each modality has unique contrast mechanisms, artifacts, and subtle anatomical variations not represented in natural image datasets. Models pre-trained on natural images may be suboptimal for medical retrieval. Effective systems rely on large, high-quality, modality-specific datasets (41–43).

In this context, three-dimensional image retrieval is particularly promising for modalities such as CT, although it remains an emerging area with limited benchmarks and standardized datasets (5).

### 3.3 Scalability and generalizability across institutions

Many systems are developed for specific diseases, organs, or protocols, limiting their usefulness outside controlled settings. In clinical practice, retrieval must function across institutions with different scanners, protocols, and populations. Vision foundation models offer potential by learning generalizable representations from large heterogeneous datasets (44, 45).

### 3.4 Definition of similarity

Similarity is not universal in radiology but inherently task-dependent and context-specific. For example, in diagnostic support, it centers on lesion morphology and confirmed histopathological diagnosis; in treatment response monitoring, on volumetric change patterns and temporal dynamics rather than static appearance; in emergency settings, on lesion location and acuity for time-critical decision-making.

Current systems rely on a single embedding space that conflates all dimensions into one metric, ignoring this multiplicity. Recent work on multi-granularity annotations derived from radiology reports has begun addressing this limitation, but no existing system allows clinicians to dynamically specify the axis of similarity at query time (46). Developing task-adaptive retrieval mechanisms remains a critical open challenge for clinical translation.

### 3.5 Multimodal approaches

As the field advances, multimodal approaches are increasingly important to bridge the semantic gap. The concept of *diagnostic orchestration*, also referred to as *superdiagnostics*, describes a paradigm in which insight emerges from coordinating heterogeneous data sources like imaging, pathology, laboratory values and clinical records, rather than isolated modalities (47).

Integrating such heterogeneous data remains challenging due to differences in data format, scale, and semantic meaning. Traditional fusion methods often fail to capture these complex relationships (48). Recent work has demonstrated the potential of graph-based modeling in which each modality is represented as a node and their relationships as edges (49, 50).

### 3.6 Computational efficiency

High-resolution images, multimodal data, and 3D studies require substantial computational resources for storage, processing, and retrieval. These demands complicate large-scale hospital deployment, particularly when models require frequent updates (51). To address these issues, retrieval systems rely on strategies such as compact feature representations, approximate nearest-neighbor search, and efficient indexing techniques, which allow rapid retrieval without exhaustive comparisons (52–55).

### 3.7 Temporal dimension

Most existing retrieval approaches focus on individual images or single examinations. However, many clinical questions are inherently longitudinal. Radiologists often assess how findings evolve over time, such as tumor growth, treatment response, or disease progression. This critical shortcoming motivates the need for a new paradigm: trajectory retrieval.

## 4 Trajectory retrieval: beyond static similarity

Clinical decision-making in radiology is inherently longitudinal. Radiologists assess not only the current state of a lesion or anatomical structure but also its temporal evolution, monitoring factors such as tumor growth, treatment response, and chronic disease progression. This temporal dimension is essential for accurate diagnosis, prognosis, and treatment planning.

Recent AI research has highlighted the value of longitudinal data integration. For example, studies such as CARDIA have shown that combining multimodal imaging across time can improve dynamic prediction models for complex diseases, including cardiovascular and renal conditions (56).

### 4.1 Trajectory modeling

The temporal dimension has attracted growing interest in medical AI, and several distinct works have emerged in recent years. Research has focused on patient trajectory modeling, where methods based on large-scale healthcare records aim to reconstruct typical disease courses and stratify patients accordingly (57, 58). Machine learning has further refined these approaches by capturing heterogeneity in individual progression patterns, supporting personalized prediction in precision medicine (59). A second, more recent line has brought longitudinal reasoning into medical imaging itself: transformer-based models have been proposed for disease prognosis from sequential scans, and generative frameworks have begun to forecast image-level disease progression (60, 61).

Despite these advances in modeling temporal dynamics, even multimodal retrieval frameworks that combine imaging with other data sources have so far treated each study as a static snapshot, without incorporating temporal evolution (62). The recent TemMed-Bench benchmark represents a first step toward trajectory retrieval, but it remains limited to pairs of images (i.e., two time points) rather than full longitudinal sequences (63).

Taken together, these efforts expose a fundamental gap. While temporal dynamics are increasingly modeled, they are not yet effectively integrated into retrieval frameworks.

### 4.2 Definition and novelty

To better reflect this reality, the concept of **trajectory retrieval** is introduced: a paradigm designed to retrieve clinically and radiologically comparable sequences of imaging studies, enriched with associated clinical data, that collectively describe the evolution of a condition over time. Trajectory retrieval enables the identification of patients or cases whose longitudinal imaging trajectories exhibit analogous morphological changes, functional trends, or therapeutic responses, thereby aligning more closely with the temporal nature of clinical practice.

The paradigm differs from prior work in three key respects. First, it is retrospective rather than predictive: while trajectory modeling generates probabilistic forecasts of a patient's future course, trajectory retrieval identifies historical cases that have already followed a similar longitudinal evolution, providing validated precedent trajectories. Second, it is longitudinal rather than static: conventional CBIR matches individual images at a single timepoint, but trajectory retrieval compares entire sequences, capturing temporal dynamics. Third, it is imaging-native and workflow-integrated: it is grounded in visual data and designed for direct integration into the radiology workflow.

Although analogous concepts of sequence-level retrieval have been applied in robotics and embodied AI, their adaptation to medical imaging remains unexplored (64, 65). The trajectory retrieval paradigm proposed here represents, to our knowledge, the first formalization of this concept for clinical radiology.

### 4.3 Key obstacles in handling longitudinal data

Incorporating the temporal dimension introduces new challenges for radiology image retrieval. These temporal-specific obstacles are summarized in Figure 1.

#### 4.3.1 Trajectory similarity

The temporal dimension redefines similarity. In longitudinal imaging, it must capture how a patient's condition evolves over time rather than focusing on visual resemblance at a single timepoint. Trajectory similarity spans morphological, temporal, and clinical dimensions. Patients with different scans at a given moment may follow similar diseases or therapy trajectories, while visually similar images may mask divergent courses. Methods such as temporal embeddings, sequence alignment, and multimodal contrastive learning have been explored to address this complexity (66).

#### 4.3.2 Lack of longitudinal ground truth

The lack of a universally accepted ground truth remains a critical barrier for longitudinal similarity. Most publicly available datasets provide image-level annotations without capturing disease evolution. Even datasets that include longitudinal imaging often lack detailed clinical labels reflecting progression or treatment response (46, 67). Human- in-the-loop (HITL) approaches offer a promising way forward by allowing radiologists to provide relevance feedback, and reinforcement learning frameworks can incorporate this feedback to iteratively improve retrieval performance (68–70).

#### 4.3.3 Loss of temporal information

Anonymization procedures frequently remove acquisition dates from DICOM files, disrupting temporal coherence needed for trajectory analysis. To mitigate this issue, de-identification methods have been proposed to retain relative temporal information, allowing for secure data sharing while preserving the integrity of longitudinal analyses (71).

## 5 Bridging the gap between research and clinical practice

Despite extensive research, clinical adoption of radiology image retrieval remains limited, mainly due to the difficulty of building large, privacy-compliant datasets with confirmed diagnoses and longitudinal follow-up. Many existing systems target narrow scenarios, and approaches based only on visual similarity often miss what truly matters clinically. Moving toward pathology-aware, multimodal, and longitudinal retrieval is challenging due to fragmented data, loss of temporal information during anonymization, and irregular follow-up.

Although methods have evolved from handcrafted features to deep learning, this progress has not yet produced broad clinical integration. Bridging this gap requires shifting from optimizing similarity scores to designing retrieval systems that mirror how diagnoses are confirmed and refined over time. Three emerging paradigms offer promising solutions. Foundation Models provide generalizable feature representations without extensive labeled data (72). Federated Learning addresses the critical bottleneck of data curation and privacy, enabling collaborative training across institutions (73). Finally, HITL offers a dynamic approach to align retrieval results with complex clinical reasoning (70).

However, each paradigm carries significant limitations. Foundation Models remain affected by documented sex and race biases in radiography, lack transparency, and are still largely unvalidated for retrieval, with high computational costs (74). Federated Learning represents an important step toward privacy-preserving collaboration but is not sufficient alone, as gradient inversion attacks may reconstruct patient-level images from shared updates, alongside challenges of communication overhead, heterogeneity, and governance (75). Similarly, RL/HITL relies on sustained expert feedback that is hard to scale and inherently subjective, risking propagation of reviewer biases into the model (76).

Overcoming these intertwined limitations will therefore require combining these complementary paradigms under rigorous multicenter validation.

### 5.1 Privacy, ethical, and regulatory considerations

The deployment of CBIR and trajectory retrieval systems also raises specific privacy, ethical, and regulatory challenges. Retrieval systems, by design, return real patient cases as output. Even when de-identified, the combination of rare findings, anatomical variants, and longitudinal sequences can increase re-identification risk, particularly in small cohorts. Trajectory retrieval amplifies this risk because linking imaging sequences across time provides richer re-identification vectors (71). Current regulatory frameworks (FDA, EU AI Act) are designed primarily for binary-output tools; CBIR systems that produce ranked reference cases occupy a regulatory gray zone (77). Systems trained on institutional data may also reflect and propagate existing biases in diagnostic practices or patient demographics, and when retrieval results influence clinical decisions, clear accountability frameworks are needed.

### 5.2 Roadmap for clinical translation

We propose a phased research agenda for clinical translation of retrieval-based systems in radiology.

The initial phase should focus on building curated, multi-institutional datasets with verified diagnoses and longitudinal follow-up, and on defining shared evaluation metrics for retrieval relevance. Building on this, a second phase should develop context-aware similarity measures, integrate foundation model embeddings into PACS, and prototype trajectory-based retrieval. Once these methodological advances reach sufficient maturity, a further phase will require multicenter prospective observer studies to assess diagnostic impact and clinical workflow, alongside HITL feedback mechanisms. A final phase will involve collaboration with regulatory bodies to clarify approval pathways, followed by pilot deployments with ongoing performance monitoring. Throughout all phases, close collaboration between AI researchers, radiologists, data curators, and regulatory stakeholders will be essential.

Together, these steps aim to help retrieval systems evolve beyond visual similarity into clinically meaningful, actionable tools that support routine radiological practice and, ultimately, better patient care.

## Data Availability

The original contributions presented in the study are included in the article/Supplementary Material, further inquiries can be directed to the corresponding author.
